# Evaluation of Three Blended Learning Courses to Strengthen Health Professionals' Capacity in Primary Health Care, Management of Sexual and Reproductive Health Services and Research Methods in Guinea

**DOI:** 10.3389/fdgth.2022.911089

**Published:** 2022-06-27

**Authors:** Tamba Mina Millimouno, Thérèse Delvaux, Jean Michel Kolié, Karifa Kourouma, Stefaan Van Bastelaere, Carlos Kiyan Tsunami, Abdoul Habib Béavogui, Marlon Garcia, Wim Van Damme, Alexandre Delamou

**Affiliations:** ^1^Maferinyah National Training and Research Center in Rural Health, Forécariah, Guinea; ^2^Department of Public Health, Institute of Tropical Medicine, Antwerp, Belgium; ^3^Belgian Development Agency (Enabel), Brussels, Belgium; ^4^Department of Clinical Sciences, Institute of Tropical Medicine, Antwerp, Belgium; ^5^Africa Center of Excellence for Prevention and Control of Transmissible Diseases (CEA-PCMT), Gamal Abdel Nasser University, Conakry, Guinea

**Keywords:** blended learning (BL), e-learning, online learning (OL), distance learning, training, health professionals (HPs), medical education, Guinea (Conakry)

## Abstract

**Background:**

Three blended courses on Primary Health Care (eSSP), Management of Sexual and Reproductive Health Services (eSSR), and Research Methods (eMR) were developed and implemented between 2017 and 2021 by the Maferinyah National Training and Research Center in Rural Health, a training and research institution of the Ministry of Health in Guinea. The study objectives were to evaluate the reasons for dropout and abstention, the learners' work behavior following the training, and the impact of the behavior change on the achievements of learners' organizations or services.

**Methods:**

We evaluated the three implemented courses in 2021, focusing on levels 3 and 4 of the Kirkpatrick training evaluation model. Quantitative and qualitative data were collected through an open learning platform (Moodle), via an electronic questionnaire, during the face-to-face component of the courses (workshops), and at learners' workplaces. Descriptive statistics and thematic analysis were performed for quantitative and qualitative data, respectively.

**Results:**

Out of 1,016 applicants, 543 including 137 (25%) women were enrolled in the three courses. Over the three courses, the completion rates were similar (67–69%) along with 20–29% dropout rates. Successful completion rates were 72% for eSSP, 83% for eMR and 85% for eSSR. Overall success rate (among all enrollees) ranged from 50% (eSSP) to 58% (eSSR). The majority (87%) of the learners reported applying the knowledge and skills they acquired during the courses through activities such as supervision (22%), service delivery (20%), and training workshops (14%). A positive impact of the training on utilization/coverage of services and increased revenues for their health facilities were also reported by some trainees.

**Conclusion:**

These findings showed fair success rates and a positive impact of the training on learners' work behavior and the achievements of their organizations.

## Introduction

In recent years, there has been increasing use of e-learning and blended learning in continuing medical education (1–4). However, comprehensive design, implementation, and impact evaluation methods are needed for possible future replication in similar settings.

E-learning can be defined as the use of information and communication technology, digital tools, or media to deliver education (5). In spite of the need for traditional skills to align teaching strategies with teaching/learning objectives, e-learning is a valuable addition to the teaching toolbox, as it provides instructional resources, activities, assessments, and feedback online (1). Blended learning combines e-learning and traditional face-to-face methods to run asynchronous and synchronous learning activities (6–10). The flexibility of blended learning enables the online delivery of content together with the best features of classroom interaction and live instruction to personalize learning, encourage thoughtful reflection, and individualize instruction across a diverse group of learners (11, 12). The increasing use of blended learning is strongly driven by three reasons – improved pedagogy – increased access and flexibility – and increased cost-effectiveness (6). The recent COVID pandemic undeniably is an additional driver at the global level for e-learning and blended learning development.

Evaluations enable us to determine whether and how well the training accomplished the assigned goals and objectives (13). Evaluation remains one of the biggest challenges for training institutions, particularly workplace training professionals, and few organizations conduct comprehensive evaluations of their training. Several reasons have been reported, such as the time between training and the opportunity to use the skill or knowledge or the challenge of evaluating training and outcomes for complex skills or problem-solving (13). Moreover, few evaluations go beyond assessing learner reaction and satisfaction, which are levels one or two of Kirkpatrick's four-level model (Reaction, Learning, Behavior, and Results), commonly used for training evaluations (14, 15). The introduction of e-learning and distance support for these training programs does not seem to have improved training evaluations (13).

In Guinea, the development of quality human resources for health, particularly in primary health care, management of sexual and reproductive health services, and research methods, is a priority for health authorities and their partners working on strengthening the health system. Over the past 10 years, internet coverage considerably increased in the whole country (0.4% in 2010 to 33% in 2018). The Ebola crisis (2014–2016) had put on hold several face-to-face training and led to develop a novel blended learning approach. Three blended learning courses were developed and implemented in Guinea. Evaluations of the first two courses (eSSP and eSSR) have already been reported (16, 17). However, these reports primarily focused on completion and success rates, factors associated with success, and learners' perceptions (reactions) of the training (levels 1 and 2 of the Kirkpatrick model) (16, 17). Moreover, reasons for dropout and abstention among training participants, the change in their work behavior (level 3), and the impact of this behavior change on the results of their organizations (level 4) were not assessed. Thus, the main focus of this study was to evaluate (1) reasons for dropout and abstention, (2) change(s) in work behavior reported by learners following the training, and (3) the impact of the work behavior change on the achievements of the organizations or services where the learners work.

## Methods

### Course Development and Implementation

The three courses were developed in French and implemented between 2017 and 2021 by the Maferinyah National Training and Research Center in Rural Health or Maferinyah Center, a training and research institution of the Ministry of Health in Guinea located in Forecariah district (50 km from Conakry). The development and implementation of the courses were funded by the Belgian Development Agency (Enabel), and the Directorate-General for Development Cooperation and Humanitarian Aid (DGD). More details on the Guinean context and the actual development of the courses have been provided earlier (16, 17). The first two courses on Primary Health Care (eSSP) and Management of Sexual and Reproductive Health Services (eSSR) addressed the local health system context. They targeted health professionals working in health facilities and institutions, as well as medical students at the end of their medical school studies, before starting to work in the field (Table 1). The third course on Research Methods (eMR) targeted medical students before completing their thesis research component at the end of the medical school and health professionals already or intending to be involved in public health and research (Table 1). The “Analyze, Design, Develop, Implement and Evaluate (ADDIE)” instructional design model was used to develop the training modules (18). The capacity to develop and implement the course was built within the Maferinyah Center team through specific courses on e-learning and continuous coaching. The three courses were delivered through an online open Learning Management System (LMS), the Moodle platform. The eSSP, eSSR, and eMR respectively counted seven, nine and six two-week online modules (Table 1). The three courses consisted of a major online component or asynchronous learning with modules that could be downloaded in “Html” format. Synchronous learning included a continuous discussion forum, a Zoom call scheduled for each module, and was initiated during the COVID-19 epidemic (with increased use for the last training cohorts), and a five-day face-to-face capacity building workshop at the end of the training for a sample of trainees (Table 1).

**Table 1:** Blended courses, modules and learning modalities in Guinea, 2018–21^*^.

**Modules**	**Primary health care (PHC)** **eSSP**	**Management of sexual and reproductive health services (SRH)** **eSSR**	**Research methods (RM)** **eMR**	**Learning modalities**
1	Introduction to PHC	Introduction to SRH	Introduction to	- Each course is delivered to a cohort of enrolled participants. - Each Module is delivered over 2 weeks, facilitated by an “expert” or e-learning team member knowledgeable in the topic. - A forum of discussion is open to all learners experts to interact; a Zoom learning session is added to each module (last cohorts) - An Information Technology specialist is available for technical questions/problems. - Face-to face capacity-building workshop is held at the end of the course.
2	Local health system	Local health system	Literature review & reference management	
3	Data management in PHC	Data management in SRH	Ethics in research	
4	Monitoring a health zone	Monitoring a health zone	Research protocol	
5	Quality and integration in PHC	Quality and integration in SRH	Conduct of research	
6	Community health	Community health		
7	Health promotion	Health promotion		
8		Gender & rights in SRH		
9		Gender-based violence		

* *Translated from French*.

### Study Design and Period

The three implemented blended courses (eSSP, eSSR and eMR) were evaluated from June to August 2021. A cross-sectional study using a mixed-methods approach was used. We collected quantitative and qualitative data in four stages: (i) through the learning platform (Moodle course statistics); (ii) via an electronic questionnaire, (iii) during the learners' capacity building workshops (opinion on short-term effect), and (iv) in the field to interview participants at their workplaces.

### Theoretical Framework for Training Evaluation

We used Kirkpatrick's four-level model (Reaction, Learning, Behavior, and Results), which is one of the widely known evaluation models adapted to education (14, 15) (Supplementary Figure 1).

#### Operational Definitions

*Completion rate* was the number of learners who completed the course by performing all learning activities over the total number of enrollees. *Dropout rate* was the number of learners who dropped out from the course after completing some activities over the total number of enrollees. *Abstention rate* was the number of enrollees who ultimately did not log into the online learning platform although they had received all necessary information to access it, over the total number of enrollees. *Successful completion rate or success rate* was the number of learners who passed the course (with an overall mean of marks greater than or equal to five out of ten), over the number of learners who completed the course. *Overall success rate* was the number of learners who passed the course, over the total number of enrollees.

### Data Collection

#### Quantitative Data Collection

We collected selected sociodemographic characteristics and results of courses (participation, completion, and success rates) for all participants in the three courses through the Moodle open platform statistics. This information aimed to provide some basic information about the course before addressing level 2 of the Kirkpatrick model. An additional electronic questionnaire (KoboToolbox) was sent to all course participants by email to better understand the behavioral changes at work following the training experience (level 3 of the Kirkpatrick training evaluation model).

#### Qualitative Data Collection During the Capacity Building Workshops

During three workshops, which took place between June and July 2021 for 5 days per workshop, time was set aside to collect data from learners who attended the different workshops. In-depth individual interviews (IDIs) and focus group discussions (FGDs) were carried out by the primary author (TMM) and JMK using an interview checklist. As participants were accommodated at the Maferinyah Center, the IDIs and FGDs were performed during the evenings and lasted around 50 and 70 min, respectively. This qualitative data collection during the workshops aimed at achieving objectives 3 and 4 of the evaluation.

Five learners per cohort were purposively selected for the workshops according to their performance levels – the first two learners on the list, one from the middle, and the last two learners (with a low score and/or not having completed the course). The choice of interviewees was purposive and respected the principle of maximum variation and the data saturation model (19).

#### Additional Qualitative Field Data Collection

A team of three people collected additional qualitative data in the field for seven days to complement the already available data, more specifically to reach levels 3 and 4 of the evaluation model. We collected data from (1) learners who were not selected for the consolidation workshops (having validated or not the courses); (2) learners in their professional context and situation. We purposively selected interviewees along the Enabel intervention axis (regions of Conakry, Kindia, and Mamou), considering the principle of maximum variation and the data saturation model (19).

### Data Analysis

For the quantitative analysis, the data was extracted from Moodle platform and Kobo Toolbox through an Excel spreadsheet and imported into SPSS version 21 for analysis. Descriptive statistics were performed as proportions for categorical variables and median with standard deviation for continuous variables.

Regarding qualitative data, the interviews recorded in French were transcribed in full. We applied thematic analysis (20, 21) to analyze the responses to open-text questions. Each open-text response was coded in NVivo (TD). Inductive coding was used with codes discussed with the research team, grouped into themes, and based on the inductive thematic saturation model (19). Qualitative data were further analyzed to identify and document connections between the themes. Quotes from the open-text responses were used to illustrate findings from themes and quantitative results.

### Ethical Considerations

The research protocol was approved by the National Ethics Committee for Health Research in Guinea (No: 022/CNERS/2020) and the ITM Institutional Review Board in Belgium (Reference Code: 1363/20). Regarding the qualitative component of the study, free, informed, and oral consent was obtained from each selected participant before carrying out the interviews. Both quantitative and qualitative data were only accessible to the research team. The database is stored on a computer protected by a password at the Maferinyah Center.

## Results

### Characteristics of Learners of the Three Blended Courses

Overall, 1016 health professionals, including 235 (23.1%) women, applied for the courses (eSSP, eSSR and eMR) through calls for applications launched online between 2017 and 2020 using professional [District.Team Guinea (22)] and social (Facebook) networks.

A total of 543 learners, including 137 (25.2%) women, were selected out of the applicants (Table 2). Overall, the majority of participants were between 30 and 40 years old (*n* = 379, 70%), male (*n* = 406, 75%), medical doctors (70%), Guinean nationals, or based in Guinea (87 and 88%, respectively). Women were more represented in the eSSR course (32%) than in the other two courses. Only 23 nurses were enrolled in the three courses, and 14 midwives participated exclusively in the eSSR course. Although the majority of participants resided in Guinea (93%), some learners resided outside Guinea (*n* = 71) (Table 2).

**Table 2:** Sociodemographic characteristics of participants to three blended courses in Primary Health Care (eSSP), Management of Sexual and Reproductive Health Services (eSSR) and Research methods (eMR), Guinea, 2018–21.

**Variables**	**eSSP course** **9 cohorts** **(***n*** = 181)**		**eSSR course** **9 cohorts** **(***n*** = 212)**		**eMR course** **4 cohorts** **(***n*** = 150)**		**Total enrollees** ***N*** **= 543**	
	***N***	**%**	***N***	**%**	***N***	**%**	***N***	**%**
**Age (years)**								
<30	28	15.4%	34	16.3%	42	28.0%	104	19.1%
30–40	129	71.2%	160	75.4%	90	60.0%	379	69.7%
> 40	24	13.2%	19	8.9%	17	11.3%	60	11.0%
**Sex**								
Male	145	80.1%	144	67.9%	117	78.0%	406	74.7%
Female	36	19.9%	68	32.0%	33	22.0%	137	25.2%
**Profession**								
Medical Doctor	131	72.3%	152	71.6%	100	66.6%	383	70.5%
Midwife	0	0%	14	6.6%	0	0%	14	2.5%
Nurse	10	5.5%	9	4.2%	4	2.6%	23	4.2%
Student (last year of medical studies)	14	7.7%	15	7.0%	28	18.6%	57	10.4%
Other^1^	26	14.3%	22	10.3%	18	12.0%	66	12.1%
**Residence**								
Guinea (capital city. Conakry)	134	74.0%	150	70.8%	133	88.7%	417	76.9%
Guinea (other regions)	13	7.2%	27	12.7%	14	9.3%	54	10.0%
Foreign country ^2^	34	18.8%	35	16.5%	3	2.0%	71	13.1%

1 *Community health workers, Pharmacists, Biologists, and Public health technicians*.

2 *Countries of residence: Benin, Burkina Faso, Burundi, Cameroon, Congo-Brazzaville, Côte d'Ivoire, DR Congo and Mali, Morocco, and Togo*.

### Main Results for the Three Blended Courses

Results of the three courses presented in Table 3 show that a similar proportion of participants completed all course modules (ranging from 67 to 69%) with dropout rates varying from 20% (eSSP) to 29% (eMR). Success rate (among those who completed the courses) ranged from 72% (eSSP) to 83% (eMR) and 85% (eSSR). Overall success rate (among all enrollees) ranged from 50% (eSSP) to 58% (eSSR). More detailed results on factors associated with successful completion rates have been described for eSSP and eSSR courses and published elsewhere (Table 3) (17).

**Table 3:** Results of learners on the blended courses in Primary Health Care (eSSP), Management of Sexual and Reproductive Health Services (eSSR), and Research methods (eMR), Guinea, 2018–21.

**Variables**	**eSSP course** **9 Cohorts** **(***n*** = 181)**		**eSSR course** **9 Cohorts** **(***n*** = 212)**		**eMR course** **4 cohorts** **(***n*** = 150)**		**Total** ***N*** **= 543**	
	***N***	**%**	***N***	**%**	***N***	**%**	***N***	**%**
Completion rate	125	69.0%	146	68.8%	101	67.3%	372	68.5%
Dropout rate	36	19.8%	46	21.6%	43	28.6%	125	23.0%
Abstention rate	22	12.1%	12	5.6%	6	04.0%	40	07.3%
Successful completion rate (*among* *learners who completed the course)*	90	72.0%	124	84.9%	84	83.1%	298	80.1%
Overall success rate (*among all enrollees*)	90	49.7%	124	58.4%	84	56.0%	298	54.8%

### Characteristics of Participants in the Evaluation of the Courses

A total of 233/543 (43%) participants completed the online questionnaire, and 53 participated in individual in-depth interviews (IDIs) and five focus group discussions (FGDs). Sociodemographic characteristics of the participants in the evaluation or respondents are in line with those of course participants (Supplementary Table 1). The majority of respondents were between 30 and 40 years of age (72.2%), with a mean age of 36 years (SD = 6.4). The majority of respondents (77.7%) were male. Most participants in the evaluation were Guineans by nationality, resided in Guinea (88.8%), and in urban areas at the time of training (88.0%). Medical doctors were the most represented (82.4%) and working full-time at the time of the training (71.2%). The majority (*n* = 211; 90.6%) of respondents had started the course, of which 163 (77.3%) had completed the course and 48 (22.7%) had dropped out (Supplementary Table 1).

### Reasons for Dropout During the Course or Not Attending After Enrolment (Abstention)

The main reasons reported for dropping out during the course (after starting at least one module) were interference with other courses or work overload (62.5%), lack of technological skills (10.4%), travel or displacement (8.3%), computer breakdown or loss (6.3%), internet connection problem (4.2%), and illness (4.2%) (Table 4). On the other hand, participants reported that the main reasons for not attending the courses after being enrolled (without even starting one module) were moving to or changing city where the internet network is not stable (31.8%), work overload (18.2%), interference with another ongoing course (18.2%), difficulty in accessing the learning platform (13.6%) and late receipt of information (13.6%) (Table 4).

**Table 4:** Reasons for course dropout and abstention.

**Reasons for dropout/ abstention**	**Dropout** **(*n* = 48)**	**%**	**Abstention** **(*n* = 22)**	**%**
Interference with other courses/high workload	30	62.5	8	36.4
Lack of technical skills	5	10.4		
Difficulty in accessing the platform			3	13.6
Computer failure/theft	3	6.3		
Travel or commuting/ Moving to or changing cities where the internet was not stable	4	8.3	7	31.8
Unable to buy the internet package			1	4.5
Illness	2	4.2		
Assignment to other jobs or locations	1	2.1		
Lack of technical support from supervisors	1	2.1		
Late information			3	13.6

### Change(s) in Work Behavior Reported by Learners Following the Training

#### Application of Acquired (New) Knowledge and Skills

The majority (86.5%) of the participants reported that they put into practice the knowledge they had acquired during the course through several activities such as supervision (22.0%), service delivery (19.9%), training workshops (14.2%), work meetings (12.8%) and teaching (10.6%) (Table 5). An eSSR learner stated that: “*During supervisions, especially integrated supervisions, I am often given the reproductive health part, as they know that I took the eSSR course. So, I calculate and interpret indicators and develop improvement plans for bottlenecks”* (EIA, eSSR, female).

**Table 5:** Application of new knowledge and skills.

**Variables**	***N***	**Percentage**
**Applied acquired knowledge (***n*** = 163)**		
Yes	141	86.5
No	22	13.5
**Activities through which the acquired knowledge was practiced (***n*** = 141)**		
Supervision	31	22.0
Service delivery	28	19.9
Training workshop	20	14.2
Working meeting	18	12.8
Teaching	15	10.6
Staff meeting at the hospital	11	7.8
Monthly report	8	5.7
Drafting research protocols/supervision of medical students' thesis	8	5.7
Monitoring of health services	2	1.4

An eMR learner asserted: “*The studies we carry out in the cardiology department, of course, require the drafting of a research protocol first of all. So, I put into practice the knowledge I acquired in the eMR course by writing research protocols and correcting students' theses, but also by writing scientific articles for publication. These activities really allow me to apply what I learned in the eMR course”* (EIA, eMR, male).

Some participants (13.5%) did not apply what they had learned because the course was not applicable to their daily work or current job.

“*I have not yet applied the knowledge and skills I have acquired, but I would like to do so in the future because currently my job is not related to public health; I am in research*” (EIA, eSSP, male).

Although they were not yet applying the acquired knowledge, other participants stated that they would continue to read the courses to keep up to date, hoping to practice what they had learned in the future.

#### Change in Behavior at Work or in Professional Practice

Participants who applied the knowledge and skills they acquired in the course reported that they had changed their work behavior, which is directly related to the course. The main findings are presented in Box 1. Many respondents reported feeling more confident and more comfortable at work following the training. The training also increased the visibility of some participants in their workplaces and was a source of inspiration for new activities in their work. For some respondents, the training allowed them to improve their professional practice and that of their colleagues in the same department. The training also enabled some participants to change their behavior in the workplace and provide support to other health services when called upon.

Box 1 **Reported behavioral outcomes and impact on work organizations/services.**

**Table:** 

**Theme**	**Quotes**
**Behavioral outcomes**	
More confidence and comfort in their work	“*Since I am in the research field, whenever there are issues to discuss, my interventions are not like before because now I reason with confidence and am at ease*” (EIA, eMR, male).
Increased visibility at work	“*Yes, of course, because before, few people entrusted me with their work (protocols and theses), but when I finished this training, we produced a protocol,l and since they saw it, many lecturers in the department entrust me with their theses to transform them into articles because they see that I am doing an excellent job, and all this thanks to this training that I followed*” (EIA, eMR, male)
Training as a source of inspiration at work	*Well, my usual professional practice is general medicine; consulting patients is everything; maybe what I can say is that for some time, I have been much more interested in reviewing the files of the consulted patients to make articles. It came to me through this course. It's something I didn't realize at first, but I'm talking to my boss about doing articles based on the patients we consult*” (EIA, eMR, female).
Training helped participants to improve not only their professional practice, but also that of their colleagues in the same department	“*Yes, there has been a net improvement not only in my professional practice but also in the professional practice of my health workers because today, although our activities are done routinely, there is a certain organization that is done at the level of the service regarding data management and even professional behavior. Many things have changed*” (EIA, eSSR, male). “*I admit that the course has influenced my way of doing things; firstly, I am the medical coordinator of an epidemic treatment center, so this course has enabled me to put forward arguments during the daily staff meetings by explaining to my medical staff the value of patient-centered care, and it is really from the module on quality of care that I got these tools; Secondly, in parallel with the coordination of the epidemic treatment center, the strategy office of the Ministry of Health often calls me for supervision missions (…) during which I analyzed the services at the deconcentrated level by collecting data and calculating indicators. Then, I identified the weaknesses that I analyzed and coached these health structures to develop improvement plans*” (FGD, eSSP, participant N° 7). “*So, as I said earlier, I took advantage of the eMR course to strengthen my ability to accompany students. Since I took the eMR course, I have become much more effective in accompanying students who are developing their thesis in medicine. Also, I have started to be a research assistant for a PhD student in epidemiology and statistics, and I feel comfortable working with her because I have been really well equipped”* (EIA, eMR, female).
**Impact on institutions**	
Impact of the training on utilization/ coverage of services, health outcomes	“*We have a health center here where I often went to help the new head of the center who came because he had difficulties with the family planning service concerning the calculation of indicators, which enabled him to present a good FP coverage. Also, during the monitoring that we are doing today, we made mistakes. It was at the prefectural technical health committee (CTPS) that these mistakes were corrected. However, the course we followed has now enabled us to go and support the other health centers, to help them with the monitoring so that when the CTPS is presented, we don't find enough mistakes”* (EIA, eSSP, male). “*Yes, that's what I was telling you; this training has enabled us to really improve the quality of the data; in any case, the monitoring indicators have improved. When we came here to the CTPS, people said, “Wait a minute, what did you do? Can you explain a bit? So I would tell them about the course I had taken*” (EIA, eSSP, male). “*Of course, in the health centers I was supervising, there were fewer deaths because the system of timely referral was well established and respected through my supervision*” (EIA, eSSP, female).
Impact on increased revenues for the health facility was also reported by trainees.	“*The revenue generated in the six months following my training has increased compared to the revenue of the previous six months, which was 26 million Guinean franc*s” (EIA, eSSR, male). “*Yes, of course, of course! It has helped to improve everything that is an indicator. For example, family planning coverage has improved because we have changed the strategies with users*” (EIA, eSSP, male).

### Impact of the Training on the Achievements of Learners' Organizations or Services

A positive impact of the training on utilization/ coverage of services and increased revenues for the health facility was also reported by trainees (Box 1). An impact of the training on public health outcomes of the organizations or services where the participants' work was reported by many participants, for instance, through improved supervision (Box 1).

## Discussion

This study documented a blended learning experience consisting of a major online component recently implemented in Guinea. Results showed that fair success rates could be achieved despite challenges learners face in a low-resource setting such as Guinea. Applied knowledge and an impact on behavior and performance in trainees' workplaces were also reported by participants following the training.

E-learning and blended learning are not yet commonly used in Africa. A recent systematic review of global health capacity building and evaluations in low- and middle-income countries (2009–2019) showed that despite a sharp recent increase in e-learning or blended courses during the last 4 years, most studies conducted in Africa documented face-to-face teaching modalities (75%) (23). The same review reported that evaluations, in general lacked standardization, especially regarding the tools, and only face-to-face initiatives were evaluated in the long term beyond the individual level (23). Many evaluations are restricted to knowledge assessment through pre-and post-tests, which are indicative but limited (24, 25).

As a result, the findings of this study will further enrich the existing literature on e-learning and blended learning, shedding light on the design, implementation, and evaluation of e-learning and blended learning programs.

We found a similar course completion rate (70%) to that of a blended learning course for building workforce capacity for effective use of health information systems in Namibia and Tanzania (73%) (26), but less than a tuberculosis course completion rate (90%) in Ethiopia (27). Nonetheless, our findings align with those of studies that highlighted avenues to enhance the completion rate on online courses, though many other studies reported lower completion and success results (28–31). The success rates among enrollees reported in our study in the three courses varying from 50 to 58% are greater than those reported by several universities specialized in e-learning in Thailand, India, the United Kingdom, and France (17–48%). As Karsenti et al. (32) reported, our results demonstrated that in the African setting where internet coverage and quality and ownership of using new technologies in education remain a challenge, fair to high success rates can be achieved in e-learning or blended learning. The requirement for obtaining good results is the deployment of e-learning efficiency conditions such as a simple, attractive, and easily accessible delivery method with user-friendly navigation; but also a variety of communication tools for synchronous and asynchronous interaction between instructors and learners, and between learners themselves. Other prerequisites or enablers are quality content, a motivating pedagogical approach with clear objectives, varied learning resources, a continuous technical and pedagogical support to learners and instructors, ethical aspects, and continuous evaluation system incorporated (32).

Completing (and dropping out from) online courses remains a significant concern (31). After starting at least one module, reasons for dropping out from e-learning or blended learning were mostly related to workload, other work issues, or lack of technical skills. Very few respondents who dropped out mentioned internet connection as a reason. However, among the respondents who were enrolled but did not attend (even one-course module), internet connection or technical issues represented a major problem, and this corroborates the studies reporting that technological limitations can act as a barrier to e-learning within a faculty and geographical context (33–35). A recent Cochrane review identified factors that impact e-learning, such as interaction and collaboration between learners and facilitators, considering learners' motivation and expectations, utilizing user-friendly technology, and putting learners at the center of pedagogy. The same review called for a better understanding of the issues related to enablers and barriers associated with e-learning, and a broader framework for making e-learning effective (36).

Capacity-building programs aim to provide the human resources of organizations with the proper knowledge that will enable them to perform their tasks and improve their skills and capabilities to innovate in their activities. Bouzguenda argues that applying acquired knowledge and skills deals with action and the validity of learning (37). About nine out of ten respondents apply the knowledge gained during the training, reflecting the relevance of course content to local capacity-building needs or, in short, the validity of the training. However, some learners (one over ten) suffer from the lack of applying the gained knowledge due to the lack of link between what they had learned and their current job or tasks; while expressing the willingness to apply the acquired knowledge in the future. This is in line with one theory of factors determining learning transfer, which advocates for a favorable work environment for employees to innovate (37).

Several systematic reviews have assessed e-learning training outcomes among health professionals compared to traditional learning and have shown similar results. Two reviews relating to nursing education reported high level of satisfaction among nurses or students (38, 39). A recent Cochrane review addressing the effectiveness of e-learning among health professionals concluded that e-learning could make little, or no difference in patient outcomes or health professionals' behaviors, skills or knowledge (40). The impact on public health results such as health service coverage and health outcomes in settings such as Guinea has been less documented. Nevertheless, e-learning offers an alternative method of education. In the Guinean context, it had the added value of training health professionals in their working sites throughout the country, avoiding useless travel costs and leaving health facilities without key human resources.

We used the Kirkpatrick model as a framework to help us with a more standardized evaluation. The widely used Kirkpatrick model represents a simple tool and language in dealing with the different outcomes of training and how information about these outcomes can be obtained as well as a practical approach for the typically complex evaluation process. Similarly, it leads to some limitations - it fails to grasp complexity of the context and related factors - it shows a certain rigidity - and the hierarchical order and importance of the different levels can be questioned (41, 42). According to Bove and Little, “many training evaluation methods, including Kirkpatrick, are insufficient because they were not created considering the types of data now available. Further, many misconceptions about the relationship between training and workplace performance (e.g., the commonly held myth that completing a task in learning automatically translates to successful performance on the job) have led to poor evaluation practices” (43). Therefore, the same authors recommend the use of evaluation and data collection methods that are appropriate for the given training context (43).

Among study strengths, we can point out that it assessed higher levels of the Kirkpatrick evaluation model that are not often addressed in training evaluation. Reasons behind abstention and dropout were explored, and the qualitative component included participants who both completed, succeeded, or did not complete the courses. As limitations, first, the relationship between outcomes and sociodemographic characteristics included in an earlier paper (17) was out of the scope of this mixed-methods paper. Second, although this study did assess the impact of the training on learners' daily professional activities, qualitative interviews were conducted among a limited number of learners, and it relied on self-report that is subject to bias (socially acceptable response); we could not interview the learners' hierarchy or colleagues to crosscheck the provided information.

## Conclusion

This evaluation showed fair success rates and a positive impact of the training on learners' work behavior and the achievements of their organizations.

## Data Availability Statement

The raw data supporting the conclusions of this article will be made available by the authors, without undue reservation.

## Ethics Statement

The studies involving human participants were reviewed and the research protocol was approved by the National Ethics Committee for Health Research in Guinea (No: 022/CNERS/2020) and the ITM Institutional Review Board in Belgium (IRB Reference Code: 1363/20). The patients/participants provided their written informed consent to participate in this study.

## Author Contributions

TM, KK, TD, and AD conceived and designed the research protocol. TM, JK, TD, and AD elaborated the evaluation tools. TM and JK collected the data (online survey, interviews and focus group discussions) and performed transcriptions. TM and TD analyzed and interpreted data and drafted the manuscript. All authors critically revised the manuscript and approved the final version before submission, and they are accountable for all aspects of the work.

## Funding

This evaluation was funded by the Belgian Development Agency (Enabel).

## Conflict of Interest

The authors declare that the research was conducted in the absence of any commercial or financial relationships that could be construed as a potential conflict of interest.

## Publisher's Note

All claims expressed in this article are solely those of the authors and do not necessarily represent those of their affiliated organizations, or those of the publisher, the editors and the reviewers. Any product that may be evaluated in this article, or claim that may be made by its manufacturer, is not guaranteed or endorsed by the publisher.
