# Supplementary material for: Evaluation of Three Blended Learning Courses to Strengthen Health Professionals' Capacity in Primary Health Care, Management of Sexual and Reproductive Health Services and Research Methods in Guinea
Source: Front Digit Health. 2022 Jun 27;4:911089. doi: 10.3389/fdgth.2022.911089 (PMC9271855; doi:10.3389/fdgth.2022.911089)
Supplement: Supplementary file 1 [file Table_1.DOCX]

**Supplementary material 2**

**Sociodemographic characteristics of respondents in the evaluation of the courses**

| **Variables** | **N (n=233)** | **Percentage** |
| --- | --- | --- |
| **Age** |  |  |
| ˂ 30 | 25 | 10,7 |
| 30 – 40 | 168 | 72,1 |
| ˃ 40 | 40 | 17,2 |
| Mean ±standard deviation | 36±6.4 | |
| **Sex** |  |  |
| Male | 181 | 77,7 |
| Female | 52 | 22,3 |
| **Nationality** |  |  |
| Guinean | 207 | 88,8 |
| Other ^1^ | 26 | 11,2 |
| **Country of residence at the time of training** |  |  |
| Guinea | 207 | 88,8 |
| Other | 26 | 11, 2 |
| **Type of residence at the time of training** |  |  |
| Urban | 205 | 88,0 |
| Rural | 28 | 12,0 |
| **Profession** |  |  |
| Physician | 192 | 82,4 |
| Nurse | 10 | 4,3 |
| Midwife | 5 | 2,1 |
| Other | 26 | 11,2 |
| **Work occupation at the time of training** |  |  |
| Full time | 166 | 71,2 |
| Part time | 67 | 28,8 |
| **Previous experience with online course** |  |  |
| Yes | 165 | 70,8 |
| No | 68 | 29,2 |
| **Course for which participants applied** |  |  |
| eSSP | 75 | 32,2 |
| eSSR | 82 | 35,2 |
| eMR | 76 | 32,6 |
| **Have initiated the course** |  |  |
| Yes | 211 | 90,6 |
| No (abstention or not even accessed once) | 22 | 7,4 |
| **Have completed the course (n=211)** |  |  |
| Yes | 163 | 77,3 |
| No (drop – out) | 48 | 22,7 |
| ^1^ Benin, Burkina Faso, Burundi, Cameroon, Congo-Brazzaville, Côte d’Ivoire, DR Congo and Togo. | | |
