# Supplementary figures and images for: Evaluation of Three Blended Learning Courses to Strengthen Health Professionals' Capacity in Primary Health Care, Management of Sexual and Reproductive Health Services and Research Methods in Guinea
Source: Front Digit Health. 2022 Jun 27;4:911089. doi: 10.3389/fdgth.2022.911089 (PMC9271855; doi:10.3389/fdgth.2022.911089)

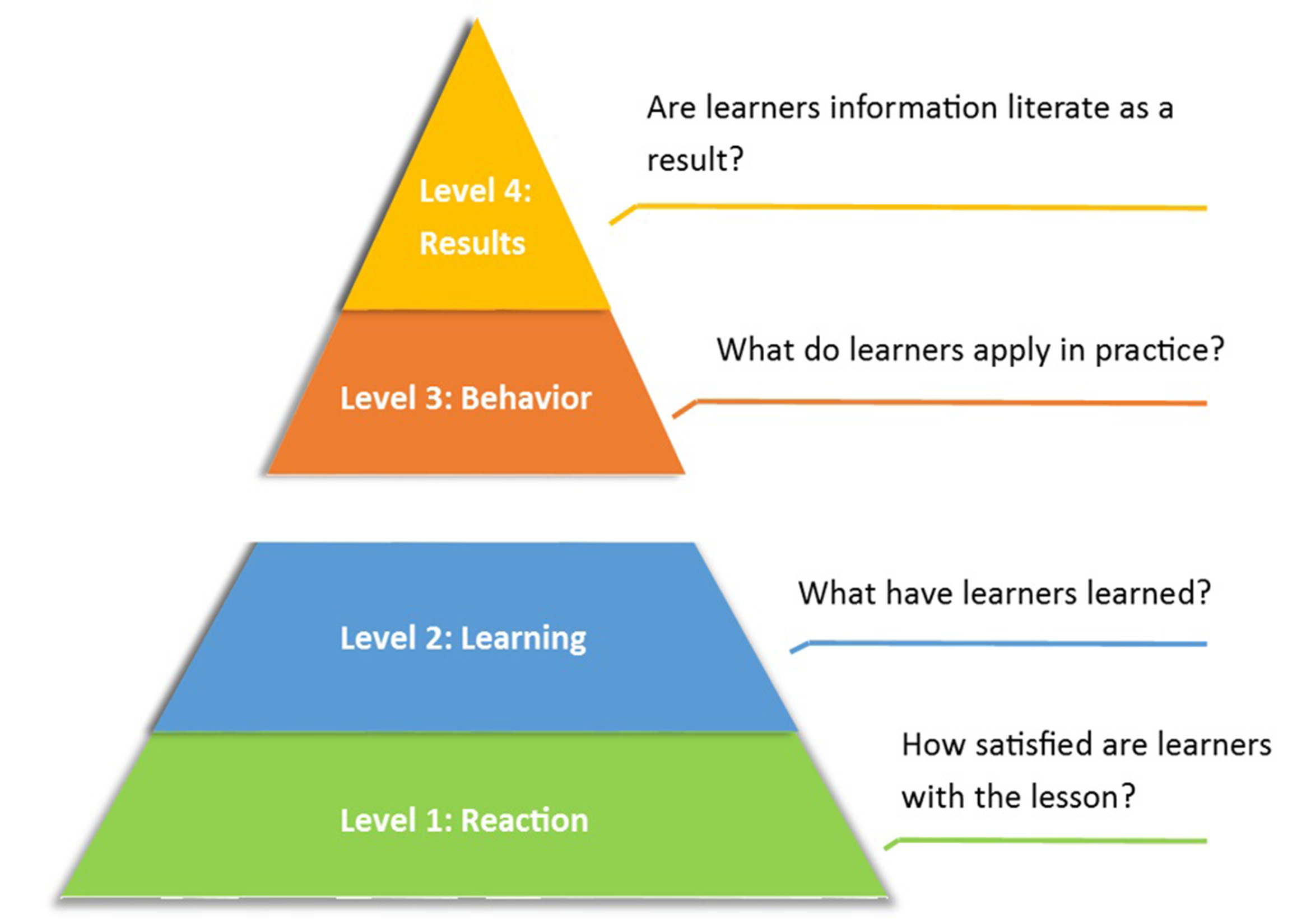

Supplement: Supplementary file 2 [file Image_1.JPG]
